# Multicentric Castleman's Disease in a Hepatitis C-Positive Intravenous Drug User: A Case Report

**DOI:** 10.1155/2011/567840

**Published:** 2011-04-11

**Authors:** D. Y. Talukder, S. N. Delpachitra

**Affiliations:** School of Medicine and Dentistry, James Cook University, Townsville QLD 4814, Australia

## Abstract

*Introduction*. We report a rare presentation of Castleman's disease in a hepatitis C-positive patient and present a short review of treatments described in other similar case reports and studies. *Case Presentation*. A 46-year-old male with untreated hepatitis C and a 16-year history of intravenous drug use presented with pleuritic chest pain and bony pain in the knee, hip, and lower back, on a background of unexplained weight loss of 40 kilograms, fevers, night sweats, and repeated infections over the last two years. Examination discovered tender hepatomegaly, a warm right knee effusion, and painless lymphadenopathy. The patient was reactive to Epstein Barr virus and cytomegalovirus; however, HIV and HHV-8 viral testing was negative. Osteomyelitis of vertebrae T8–T11 and septic arthritis of the knee were found on investigation. A lymph node biopsy revealed histology suggestive of plasmacytic Castleman's disease. The patient is to commence rituximab treatment. *Conclusion*. Castleman's disease continues to present in novel ways, which may lead to difficulties in clinicopathologic diagnosis. A growing body of evidence suggests larger studies are required to determine the best treatment for multicentric Castleman's disease, particularly in patients with a concomitant disease, including hepatitis C.

## 1. Introduction

Castleman's disease, formally known as angiofollicular lymphoid hyperplasia, is a term given to a group of disorders characterised by lymph node hyperplasia—a result of abnormal proliferation of B lymphocytes and plasma cells. 

Since its initial discovery by Benjamin Castleman in 1956, few cases of this disease have been reported. Given the rarity of the disease within the population, exact statistics on incidence and prevalence are not well known. However, from observation of case studies, it appears that the localised unicentric form of Castleman's disease is more prevalent than the multicentric type [1]. 

The aetiopathological association between multicentric, plasmacytic Castleman's disease and human herpesvirus 8 (HHV-8) is well known [1]. However, the exact cause of Castleman's disease remains speculative. Current theories include (1) a response to chronic nonspecific inflammatory processes, (2) stimulation from chronic viral infections, such as HIV, Epstein Barr virus, and HHV-8, (3) angiogenic factors leading to the characteristic increased vascularity in Castleman's disease histology, and (4) cytokine stimulation resulting in plasma cell proliferation [1]. 

Both clinical and histopathological classifications exist for Castleman's disease. Clinically, Castleman's disease is classified into unicentric and multicentric types. Unicentric Castleman's disease (UCD) generally presents with a solitary slow-growing lymph node in the mediastinum, without elevation of interleukin-6 (IL-6) and C-reactive protein (CRP). Because unicentric Castleman's disease lacks the release of inflammatory mediators, presentation of the disease is mainly due to mass effect of the lesion. In contrast, multicentric Castleman's disease (MCD) is characterised by widespread lymphadenopathy with or without hepatomegaly, and with an elevation of acute phase reactants IL-6 and CRP. Thus, MCD may present with additional constitutional symptoms including fever, hepatomegaly, anaemia, weight loss, and loss of appetite, primarily through the action of IL-6 [2]. Lymphoproliferation generally localises in the mediastinum and lung hila; however, cases of extrathoracic distribution have been documented in a number of sites, including the parotid glands, retroperitoneum, and eye [3–5].

Histologically, there are three classifications of Castleman's disease. Hyaline vascular type shows poorly formed, dysplastic lymph node germinal centres of CD21+ dendritic cells, with outer concentric layers of CD20+ lymphocytes, and associated vascularity and hyperplasia of endothelium. Plasmacytic type, in comparison, presents with layers of plasma cells in the interfollicular areas, with a larger mantle zone. The mixed variant is a combination of the aforementioned histological types. Interestingly, the hyaline vascular type is more commonly seen in UCD, and plasmacytic type is more commonly seen in MCD [6].

From the limited data available, the prognosis appears positive for UCD but poor for MCD [7]. MCD may exist as benign disease, or premalignant disease which may transform into Hodgkin's lymphoma—a marker of poorer outcome [8].

## 2. Case Presentation

A 46-year-old man presented to the emergency department with pleuritic chest pain, a swollen and painful right knee, and weight-bearing difficulty. Accompanying this was hip and back pain after a fall. This occurred on a background of unexplained progressive weight loss of 40 kilograms over the past 2 years, poor appetite, fever and drenching night sweats, increased susceptibility to infection, and intravenous drug use. He had a 16-year history of hepatitis C (HCV; genotype 3), which remained untreated due to noncompliance and poor clinic attendance. He had been a well-built professional boxer of 96 to 100 kilograms for most of his adult life; however, his admission weight was 60.61 kilograms. This was a loss of approximately 63% of his total body weight. His relevant medical history includes postoperative pulmonary embolism, disc prolapse, mild depression, and a family history of adult polycystic kidney disease. The relevant surgical history includes osteomyelitis and amputation of the third digit of the right hand, and multiple fracture repairs. Systems review was unremarkable. He has a complicated social history including a troubled upbringing, intravenous heroin use up to 1 gram per day, experimentation with most illicit drugs, a significant criminal history, and participation in an opiate substitution programme. He does not smoke tobacco or drink alcohol. 

On examination, the patient appeared acutely unwell, although vital signs were normal. He appeared cachectic, had sunken eyes, reduced skin turgor, poor dentition, extensive tattoos, and had track marks in the cubital fossae and groin. There was rib and mediastinal tenderness with no masses. Right upper quadrant abdominal tenderness was noted on palpation, with hepatomegaly 6 centimetres below the costal margin. Nontender lymphadenopathy was present in the upper left jugular nodes and bilaterally in the groin. A warm, right knee effusion was also apparent. There were no signs suggestive of infective endocarditis or chronic liver disease. Respiratory, cardiovascular, thyroid, and neurological examinations were unremarkable. 

Initial blood biochemistry revealed a noncritical normocytic normochromic anaemia (haemoglobin 102 g/L), raised CRP, elevated calcium (2.57 mmol/L), and low albumin (23 grams/L). A thyroid function test detected 18 picomols/L of free T4 (normal range 7–17 pmols/L). This was assumed to be a transient reactive elevation rather than the cause of such significant weight loss. Liver function tests, blood cultures, electrolytes, renal function, coagulation profile, *α*-fetoprotein, vasculitic screen, iron studies, vitamin B12, urate, and globulins were all within normal ranges. Hepatitis C serology was reactive with an IgG response. forth generation antigen/antibody assay was performed twice, four weeks apart, to determine HIV positivity; however, both tests returned negative. The patient's history revealed that previous HIV tests were also negative, and as such a Western blot analysis was not performed. Hepatitis B serology was consistent with immunisation. Polymerase chain reaction found the patient to be reactive to cytomegalovirus (CMV), and Epstein Barr virus (EBV), but human herpes virus 8 was not detected. Although blood IL-6 levels could have been used as a marker of systemic inflammation, C-reactive protein was chosen instead as per hospital policies. A computerized tomography of the chest and abdomen found enlarged para-aortic lymph nodes and lytic lesions in T8 and T9. There was no splenomegaly, and the liver architecture was unchanged. 

A computerized tomography pulmonary angiogram, chest X-ray, and cardiac troponin I were requested to exclude pulmonary embolism, rib fracture and pneumothorax, and myocardial infarction. All were normal. The right knee joint was aspirated, and fluid analysis showed methicillin sensitive *Staphylococcus aureus* which was treated as septic arthritis with intravenous flucloxacillin and vancomycin. Following spiking fevers over the next few weeks in hospital, a transoesophageal echocardiogram was performed to exclude infective endocarditis from a septic arthritis-induced bacteraemia. 

At this point, the patient was clinically suspected to have lymphoma or multiple myeloma, and further investigations undertaken were to exclude either. *β*-2 microglobulin and LDH levels were normal, no monoclonal band was detected on serum and urine protein electropheresis, and flow cytometry showed normal subpopulations of B cell and T cell lineages. A bone scan showed further destructive thoracic lesions but did not confirm its extent. Magnetic resonance imaging confirmed T8 to T11 osteomyelitis with epidural involvement indenting on, but not compressing, the spinal cord. Lymphoma and multiple myeloma were excluded by bone marrow biopsy. 

The diagnosis of multicentric plasmacytic Castleman's disease was histologically confirmed by an excision biopsy from an inguinal lymph node. Macroscopically, the specimen was a circumscribed ovoid nodule measuring 30 × 22 × 10 mm with a central blackish area. Microscopically, no malignant cells, Reed Sternberg cells, or their variants were seen. This was confirmed by two separate laboratories. Also noted were distinct hyaline deposits, prominent paracortical expansion by sheets of plasma cells, and subcapsular and interfollicular neutrophilic bodies. HHV-8 was not detected in this sample. 

A four-week rituximab regime was chosen as the primary treatment for this patient. Steroid treatment was also being considered as adjunct therapy.

## 3. Discussion

This case report of multicentric Castleman's disease highlights a number of emerging diagnostic and management issues with this condition. This patient was negative for both HIV and HHV-8, which emphasises the importance of including MCD as a differential diagnosis in a patient who presents with lymphadenopathy and constitutional symptoms, despite lacking two major aetiological risk factors for the disease. Even with histological evidence, the diagnosis of MCD remains inaccurate; clinicopathologically, MCD may present very similarly to other lymphoproliferative disorders [9], and as such, the presence of MCD as a separate clinical entity must always be considered.

In addition to diagnostic difficulties, this case underscores the growing need for randomised trials for suggested treatments in Castleman's disease, and to develop a clearer indication of the efficacy of each treatment regimen. A number of curative therapies for MCD are currently available and have demonstrated definite clinical improvement and remission. These include cytoreductive chemotherapy, immune modulators including steroids and interferon-*α*, and monoclonal antibodies anti-IL-6 and rituximab (for full review, see [1]). However, each has only been evidenced in few case reports, and as such the exact efficacy and use in specific clinical scenarios requires elucidation. 

The use of rituximab has been recommended for use in HIV- and HHV-8-negative patients [1]; however, no studies have investigated its role in HCV-positive cases. No clear guidelines have been made with regards to treatment of MCD in cases with associated active hepatitis C. Three previous case reports discuss presentations of concomitant treated HCV and MCD [10–12]. Interestingly, in one case report, interferon-*α*, a treatment drug for hepatitis C, was also successfully implemented as long-term therapy for concomitant MCD [12]. Although this initially led to the idea of a possible causative relationship between MCD and HCV, two separate case studies have shown promising results of interferon-*α* for MCD even in HCV- negative patients [13, 14]. 

There is evidence to suggest an association between HCV and MCD, and this may be mediated by the cytokine IL-6. IL-6, as regarded in current aetiopathological theories of MCD, is thought to stimulate plasma cell proliferation. Untreated HCV often results in a systemic inflammatory state; this may be responsible for elevated IL-6 which could be a contributing factor to the development of MCD. The existence of such a link in further research may introduce the possibility for novel uses of hepatitis C treatment as adjunct therapy for combined cases of hepatitis C and MCD. Irrespective of whether this causation exists, we speculate that, although not utilised in this patient, use of interferon-*α* therapy in cases of concomitant hepatitis C and MCD may be a useful treatment for future similar cases.

## 4. Conclusion

This case report contributes to a growing body of evidence which demonstrates the need for further study into management options for virus-associated multicentric Castleman's disease. We conclude that future research directions should be aimed at clarification of guidelines for existing management of MCD and, additionally, to determine a possible aetiopathological association between HCV and MCD.
